# Supplementary material for: Effect of multiple micronutrient-fortified bouillon on micronutrient status among women and children in the Northern Region of Ghana: Protocol for the Condiment Micronutrient Innovation Trial (CoMIT), a community-based randomized controlled trial
Source: PLoS One. 2024 May 6;19(5):e0302968. doi: 10.1371/journal.pone.0302968 (PMC11073681; doi:10.1371/journal.pone.0302968)
Supplement: S1 Table — (DOCX) [file pone.0302968.s001.docx]

**S1 Table. Table of primary outcomes**

| **Physiological group** | **Primary outcomes** |
| --- | --- |
| Non-pregnant, non-lactating women of reproductive age (WRA) | - Change in micronutrient status after 38 weeks:   - Total body vitamin A stores and liver vitamin A concentration (measured by retinol isotope dilution)   - Plasma ferritin and soluble transferrin receptor (sTfR) concentration, and calculated body iron stores (BIS)   - Plasma zinc concentration   - Erythrocyte folate concentration   - Plasma vitamin B12 concentration   - Change in hemoglobin (Hb) concentration after 38 weeks |
| Children 2-5 y | - - Change in micronutrient status after 38 weeks:   - Plasma retinol binding protein (RBP) concentration   - Plasma ferritin and soluble transferrin receptor (sTfR) concentration, and calculated body iron stores (BIS)   - Plasma zinc concentration   - Plasma folate concentration   - Plasma vitamin B12 concentration   - Change in hemoglobin concentration after 38 weeks |
| Non-pregnant, lactating women | - Change in breast milk vitamin A concentration after 12 weeks (controlling for milk fat content) - Change in breast milk vitamin B12 concentration after 12 weeks |
