# Supplementary material for: Effect of multiple micronutrient-fortified bouillon on micronutrient status among women and children in the Northern Region of Ghana: Protocol for the Condiment Micronutrient Innovation Trial (CoMIT), a community-based randomized controlled trial
Source: PLoS One. 2024 May 6;19(5):e0302968. doi: 10.1371/journal.pone.0302968 (PMC11073681; doi:10.1371/journal.pone.0302968)
Supplement: S2 Table — (DOCX) [file pone.0302968.s002.docx]

**S2 Table. Table of secondary outcomes**

| **Target group** | **Secondary outcomes** |
| --- | --- |
| Household | - Household consumption of bouillon (total and per capita) - Adherence (consumption of study-provided bouillon, expressed as % of total household bouillon consumption) |
| Non-pregnant, non-lactating women of reproductive age | - Change in serum or plasma retinol and retinol binding protein (RBP) concentration after 38 weeks - Change in serum folate concentration after 38 weeks - Change in urinary iodine concentration after 38 weeks - Urinary sodium and potassium concentration, and sodium:potassium ratio - Change in prevalence of micronutrient deficiency after 38 weeks - Change in prevalence of anaemia after 38 weeks - Inflammation (concentration of C-reactive protein (CRP) and alpha-1-acid glycoprotein (AGP) and prevalence of elevated values, and other markers including amyloid A, monocyte chemoattractant protein-1 (MCP-1), IL-6, IL-10, IL1beta, and adiponectin at baseline and endline - Current or recent malaria (by rapid diagnostic test; RDT) at baseline and endline - Morbidity (cumulative days of symptoms over study duration) - Dietary intake of bouillon, salt, fortifiable foods, energy, macronutrients, and micronutrients - Blood pressure and hypertension prevalence |
| Children 2-5 y | - Change in urinary iodine concentration after 38 weeks - Change in prevalence of micronutrient deficiency after 38 weeks - Change in prevalence of anaemia after 38 weeks - Change in plasma retinol concentration after 38 weeks - Inflammation (concentration of C-reactive protein (CRP) and alpha-a-acid glycoprotein (AGP) and prevalence of elevated values) at baseline and endline - Current or recent malaria (by RDT) at baseline and endline - Faecal calprotectin concentration at baseline and endline - Faecal microbiota at baseline and endline - Morbidity (cumulative days of symptoms over study duration) - Dietary intake of bouillon, salt, fortifiable foods, energy, macronutrients, and micronutrients - Anthropometric measurements (change in height-for-age and weight-for-height Z-scores; prevalence of stunting and wasting) - Change in Malawi Development Assessment Tool (MDAT) scores in 4 domains (gross motor, fine motor, language, social) after 38 weeks - Change in Early Years Toolbox (EYT) scores after 38 weeks |
| Non-pregnant, lactating women | - Change in prevalence of low milk nutrient concentration after 12 weeks - Dietary intake of bouillon, salt, fortifiable foods, energy, macronutrients, and micronutrients - Morbidity (cumulative days of symptoms over study duration) |
