# Supplementary material for: Effect of multiple micronutrient-fortified bouillon on micronutrient status among women and children in the Northern Region of Ghana: Protocol for the Condiment Micronutrient Innovation Trial (CoMIT), a community-based randomized controlled trial
Source: PLoS One. 2024 May 6;19(5):e0302968. doi: 10.1371/journal.pone.0302968 (PMC11073681; doi:10.1371/journal.pone.0302968)
Supplement: S4 Table — (DOCX) [file pone.0302968.s004.docx]

**S4 Table. Table of specification and definitions of hemoglobin and micronutrient biomarker variables for non-pregnant, non-lactating women of reproductive age**^1^

| **Outcome variables** | **Definitions of dichotomous outcome variables** |
| --- | --- |
| Hemoglobin (Hb; g/L) |  |
| Anemia | Hb < 120 g/L (WHO, 2011) |
| Mild anemia | Hb 110-119 g/L |
| Moderate to severe anemia | Hb 80-109 g/L |
| Severe anemia | Hb < 80 g/L |
| Hematocrit (Hct; L/L) |  |
| Anemia | Hct < 0.36 L/L (World Health Organization) |
| Plasma ferritin (µg/L) |  |
| Iron deficiency (low plasma ferritin concentration) | Plasma ferritin < 15 µg/L (WHO, 2011) |
| Plasma soluble transferrin receptor concentration (sTfR; mg/L) |  |
| Elevated plasma sTfR concentration | Plasma sTfR > 8.3 mg/L (Erhardt et al., 2004) |
| Any iron deficiency | Plasma ferritin <15 µg/L and/or sTfR > 8.3 mg/L |
| Iron deficiency anemia | (Plasma ferritin <15 µg/L and/or sTfR > 8.3 mg/L) and (Hb < 120 g/L or Hct < 0.36 L/L) |
| Low ferritin and anemia | Plasma ferritin <15 µg/L and (Hb < 120 g/L or Hct < 0.36 L/L) |
| Body iron stores (BIS; mg/kg) |  |
| Low BIS | BIS < 0 mg/kg (Cook et al., 2003) |
| Plasma zinc concentration (µg/dL) |  |
| Low plasma zinc concentration | Plasma zinc < 70 µg/dL (fasting); < 66 µg/dL (non-fasting) (Brown et al., 2004) |
| Liver vitamin A concentration |  |
| Low liver vitamin A concentration | < 0.07 µmol/g liver and < 0.1 µmol/g liver |
| Serum (plasma) retinol concentration (µmol/L) |  |
| Low retinol | Serum (plasma) retinol < 0.70 µmol/L (WHO, 2011) |
| Marginal retinol | Serum (plasma) retinol < 1.05 µmol/L |
| Plasma retinol binding protein concentration (RBP; µmol/L)^2^ |  |
| Low RBP | Study-specific cut-off to be determined |
| Marginal RBP | Study-specific cut-off to be determined |
| Serum folate concentration (nmol/L) |  |
| Low serum folate | < 10 nmol/L (Bailey et al., 2015) |
| Erythrocyte (RBC) folate concentration |  |
| Low RBC folate | < 340 nmol/L (Bailey et al., 2015) |
| Low folate and anemia | RBC folate < 340 nmol/L or serum folate < 10nmol/L and anemia (Hb < 120 g/L) |
| Plasma vitamin B12 concentration (pmol/L) |  |
| Low vitamin B12 | < 148 pmol/L (Allen et al., 2018) |
| Depleted vitamin B12 | < 221 pmol/L |
| Low B12 and anemia | Vitamin B12 < 148 pmol/L and anemia (Hb < 120 g/L) |

^1^Hb, hemoglobin; Hct, haematocrit; BIS, body iron stores; RBC, red blood cell; RBP, retinol binding protein; sTfR, soluble transferrin receptor. Procedures for adjusting micronutrient biomarkers for inflammation and methodological factors, as appropriate, will be described in the Statistical Analysis Plan. ^2^There is currently no internationally established cut-off for RBP that reflects a plasma retinol concentration of < 0.70 µmol/L or < 1.05 µmol/L, and it has been recommended that trials determine the relationship between retinol and RBP in a sub-sample of the trial population to establish study-specific RBP cut-offs.
