# Supplementary material for: Effect of multiple micronutrient-fortified bouillon on micronutrient status among women and children in the Northern Region of Ghana: Protocol for the Condiment Micronutrient Innovation Trial (CoMIT), a community-based randomized controlled trial
Source: PLoS One. 2024 May 6;19(5):e0302968. doi: 10.1371/journal.pone.0302968 (PMC11073681; doi:10.1371/journal.pone.0302968)
Supplement: S3 File — (DOCX) [file pone.0302968.s007.docx]

**S5. Supplemental Methods: Inclusion, Exclusion, and Deferral Criteria**

**Inclusion/exclusion criteria at recruitment (home visit):**

Household

Inclusion criteria are:

1. Head of household provides oral consent for the participation of household members (index participants), and willingness to have study-provided bouillon cubes used in their household cooking for the next 10 months;

Exclusion criteria are:

1. Reported chronic medical condition requiring frequent blood transfusion (e.g. severe forms of thalassemia) among any household members;
2. Current participation of any household member in a clinical trial;
3. Reported shrimp, wheat, milk, soy, eggs, celery, fish, or mollusk allergy, or a previous adverse reaction to bouillon by the participant or any member of their household.

Non-pregnant, non-lactating women of reproductive age (WRA)

Inclusion criteria are:

1. Non-pregnant non-lactating women of reproductive age (15 - 49 years);
2. Signed the informed consent form (or in the case of adolescents 15-17 years of age, unmarried and still living with their parents, assent provided from the index participant and consent from a parent or guardian);
3. Planning to remain in the study area for the next 10 months;
4. Willing to use study-provided bouillon in household cooking for the next 10 months;
5. Not planning to become pregnant during the next 10 months.

Potential participants will be excluded if any of the following apply:

1. Severe illness warranting immediate hospital referral;
2. COVID-19 diagnosis or exposure^[[1]](#footnote-1)^ in the previous two weeks [individual may repeat eligibility assessment once after a deferral period of at least 2 weeks];
3. Presence of morbidity symptoms suggesting COVID-19 infection (fever [temperature > 38°C], chills/shaking, dry cough, shortness of breath or difficulty breathing, loss of smell or taste within the past 72 hours) [individual may repeat eligibility assessment once after a deferral period of at least 2 weeks];
4. Chronic severe medical condition (e.g. malignancy) or congenital anomalies requiring frequent medical attention or potentially interfering with nutritional status;
5. Unable to provide informed consent due to impaired decision-making abilities.

Children 2-5 years of age (24-59 mo)

Inclusion criteria are:

1. Child 2-5 years of age (24-59 mo);
2. Signed informed consent for the child’s participation from a parent or guardian;
3. Planning to remain in the study area for the next 10 months;
4. Caregiver willing to use study-provided bouillon in household cooking for the next 10 months.

Potential participants will be excluded if any of the following apply:

1. Severe illness warranting immediate hospital referral;
2. COVID-19 diagnosis or exposure in the previous two weeks [individual may repeat eligibility assessment once after a deferral period of at least 2 weeks];
3. Presence of morbidity symptoms suggesting COVID-19 infection (fever, [temperature > 38°C], chills/shaking, dry cough, shortness of breath or difficulty breathing, loss of smell or taste within the past 72 hours) [individual may repeat eligibility assessment once after a deferral period of at least 2 weeks];
4. Mid-upper arm circumference (MUAC) < 11.5 cm;
5. Chronic severe medical condition (e.g. malignancy) or congenital anomalies requiring frequent medical attention or potentially interfering with nutritional status.

Lactating women

Inclusion criteria are:

1. Non-pregnant women of reproductive age (15 - 49 years); currently breastfeeding a child who is 4-18 months of age;
2. Signed the informed consent form (or in the case of adolescents 15-17 years of age, unmarried and still living with their parents, provide assent from the index participant and consent from a parent or guardian);
3. Planning to remain in the study area for the next 4 months;
4. Planning to breastfeed for the next 4 months;
5. Willing to use study-provided bouillon in household cooking for the next 4 months;
6. Not planning to become pregnant during the next 4 months.

Potential participants will be excluded if any of the following apply:

1. Pregnancy (determined by self-report);
2. Severe illness warranting immediate hospital referral;
3. COVID-19 diagnosis or exposure in the previous two weeks [individual may repeat eligibility assessment once after a deferral period of at least 2 weeks];
4. Presence of morbidity symptoms suggesting COVID-19 infection (fever[temperature > 38°C], chills/shaking, dry cough, shortness of breath or difficulty breathing, loss of smell or taste within the past 72 hours) [individual may repeat eligibility assessment once after a deferral period of at least 2 weeks];
5. Chronic severe medical condition (e.g. malignancy) or congenital anomalies requiring frequent medical attention or potentially interfering with nutritional status;
6. Unable to provide informed consent due to impaired decision-making abilities.

**Exclusion criteria at baseline screening visit (WRA only):**

Non-pregnant, non-lactating women of reproductive age

Potential participants will be excluded if any of the following apply:

1. Hemoglobin < 80 g/L **at baseline screening visit**;
2. Severe illness warranting immediate hospital referral;
3. COVID-19 diagnosis or exposure in the previous two weeks [individual may repeat eligibility assessment once after a deferral period of at least 2 weeks];
4. Presence of morbidity symptoms suggesting COVID-19 infection (fever [temperature > 38°C], chills/shaking, dry cough, shortness of breath or difficulty breathing, loss of smell or taste within the past 72 hours) [individual may repeat eligibility assessment once after a deferral period of at least 2 weeks];
5. Recent diarrhea [≥3 liquid or semiliquid stools in 72 hours]) [individual may repeat eligibility assessment once after a deferral period of at least 2 weeks];
6. Reported consumption of vitamin A-rich foods (e.g., liver) in the previous 24 hours [individual may repeat eligibility assessment once after a deferral period of at least 2 weeks];
7. Pregnancy (as ascertained via urine pregnancy test for human chorionic gonadotropin**,** HCG, on the day of isotope dosing);
8. Incomplete consumption of vitamin A isotope dose;
9. Positive malaria RDT on the day of isotope dosing [individual may repeat eligibility assessment once after a deferral period of at least 2 weeks];
10. CRP > 5 mg/L on the day of isotope dosing [individual may repeat eligibility assessment once after a deferral period of at least 2 weeks].

**Exclusion criteria at baseline visit:**

Non-pregnant, non-lactating women of reproductive age

1. Hemoglobin < 80 g/L;
2. Severe illness warranting immediate hospital referral;
3. COVID-19 diagnosis or exposure in the previous two weeks [individual may repeat eligibility assessment once after a deferral period of at least 2 weeks];
4. Presence of morbidity symptoms suggesting COVID-19 infection (fever [temperature > 38°C], chills/shaking, dry cough, shortness of breath or difficulty breathing, loss of smell or taste within the past 72 hours) [individual may repeat eligibility assessment once after a deferral period of at least 2 weeks];
5. Pregnancy (determined by self-report).

Children 2-5 years of age

1. Hemoglobin < 70 g/L;
2. Severe illness warranting immediate hospital referral;
3. COVID-19 diagnosis or exposure in the previous two weeks [individual may repeat eligibility assessment once after a deferral period of at least 2 weeks];
4. Presence of morbidity symptoms suggesting COVID-19 infection (fever [temperature > 38°C], chills/shaking, dry cough, shortness of breath or difficulty breathing, loss of smell or taste within the past 72 hours) [individual may repeat eligibility assessment once after a deferral period of at least 2 weeks];
5. Severe acute malnutrition at baseline (weight-for-height Z-score < -3 SD or bilateral oedema).

Lactating women

1. Severe illness warranting immediate hospital referral;
2. COVID-19 diagnosis or exposure in the previous two weeks [individual may repeat eligibility assessment once after a deferral period of at least 2 weeks];
3. Presence of morbidity symptoms suggesting COVID-19 infection (fever [temperature > 38°C], chills/shaking, dry cough, shortness of breath or difficulty breathing, or loss of taste or smell within the past 72 hours) [individual may repeat eligibility assessment once after a deferral period of at least 2 weeks];
4. Pregnancy (determined by self-report);
5. Cessation of lactation, or planning to discontinue breastfeeding in the next three months.

**Exclusion criteria during course of the intervention:**

Non-pregnant, non-lactating women of reproductive age

1. Pregnancy (determined by self-report);
2. Pregnancy (as ascertained via urine pregnancy test for human chorionic gonadotropin at the endline 1 visit [pre-endline isotope dosing]).

Lactating women

1. Cessation of lactation (determined by self-report);
2. Pregnancy (determined by self-report).

**Note:** If a COVID-19 exposure or diagnosis, or if COVID-19-related morbidity symptoms are reported during biweekly visits during the intervention trial, the participant will be allowed to remain enrolled, but field staff will follow procedures for “no contact” study activities (e.g., “no contact” bouillon ration drop-off) for a 2-week period.

**Deferral criteria for endline assessments**

Replicate endline assessments (3 weeks apart for blood biomarkers and 1 week apart for breast milk biomarkers) will be conducted for each of the primary outcomes except estimation of total body vitamin A stores among women. For all participants, each endline assessment will be deferred if any of the following apply:

1. COVID-19 exposure or positive test in the previous two weeks [individual may repeat eligibility assessment once after a deferral period of at least 3 weeks for WRA and children or at least 2 weeks for lactating women];
2. Presence of morbidity symptoms suggesting COVID-19 infection (fever [temperature > 38°C], chills/shaking, dry cough, shortness of breath or difficulty breathing, or loss of taste or smell within the past 72 hours) [individual may repeat eligibility assessment once after a deferral period of at least 3 weeks for WRA and children or at least 2 weeks for lactating women].

For a participant who has both endline visits deferred, the maximum duration of participation in the study would be 44 weeks (that is, if Endline 1 is deferred from 35 weeks to 38 weeks and Endline 2 is deferred from 41 weeks to 44 weeks). In this case the household would continue to receive their usual supply of study bouillon cubes until the participants complete the Endline 2 visit. Based on pilot data we expect this situation to be rare.

Note: If a non-pregnant, non-lactating WRA has a hemoglobin concentration < 80 g/L, or a 2-5 year old child has a hemoglobin concentration < 70 g/L or a WHZ < -3 SD at the first endline visit, participants may remain in the trial, but will not participate in the second endline blood sample.

**Additional** exclusion criteria for pre-endline isotope dose (WRA only):

Non-pregnant, non-lactating women of reproductive age

The pre-endline vitamin A isotope dose will be administered after the endline 1 blood sample. The isotope dose will not be given if any of the following apply:

1. Hemoglobin < 80 g/L

**Additional** deferral criteria for pre-endline isotope dose (WRA only):

Non-pregnant, non-lactating women of reproductive age

The pre-endline vitamin A isotope dose will be administered after the endline 1 blood sample. The isotope dose will be deferred if any of the following apply:

1. Recent diarrhea [≥3 liquid or semiliquid stools in 72 hours]) [individual may repeat eligibility assessment once after a deferral period of at least 3 weeks];
2. Reported consumption of vitamin A-rich foods (e.g., liver) in the previous 24 hours [individual may repeat eligibility assessment up once after a deferral period of at least 3 weeks];
3. Positive malaria RDT on the day of isotope dosing [individual may repeat eligibility assessment once after a deferral period of at least 3 weeks];
4. CRP > 5 mg/L on the day of isotope dosing [individual may repeat eligibility assessment once after a deferral period of at least 3 weeks].

1. Exposure based on the WHO definition: “A contact is defined as anyone who had direct contact or was within 1 metre for at least 15 minutes with a person infected with the virus that causes COVID-19.” <https://www.who.int/news-room/q-a-detail/coronavirus-disease-covid-19-contact-tracing>. Diagnosis may be based on positive test or diagnosis by a medical professional based on clinical symptoms. [↑](#footnote-ref-1)
