# Supplementary material for: Effect of multiple micronutrient-fortified bouillon on micronutrient status among women and children in the Northern Region of Ghana: Protocol for the Condiment Micronutrient Innovation Trial (CoMIT), a community-based randomized controlled trial
Source: PLoS One. 2024 May 6;19(5):e0302968. doi: 10.1371/journal.pone.0302968 (PMC11073681; doi:10.1371/journal.pone.0302968)
Supplement: S4 File — (DOCX) [file pone.0302968.s008.docx]

Inclusivity in global research

PLOS’ policy on inclusivity in global research aims to improve transparency in the reporting of research performed outside of researchers’ own country or community and ensures that PLOS publications reporting global research adhere to high standards for research ethics and authorship. Authors of relevant research articles may be asked to complete the questionnaire below, which outlines ethical, cultural, and scientific considerations specific to inclusivity in global research. This questionnaire may be requested when researchers have travelled to a different country to conduct research, if research uses samples collected in another country, research with Indigenous populations or their lands, or if research is on cultural artefacts. Researchers travelling to another country solely to use laboratory equipment will not normally be required to complete the questionnaire. However, the questionnaire can be requested at the journal’s discretion for any submission – if you have been requested to complete this questionnaire by the PLOS journal you submitted to, please do so.

Please complete the questionnaire below and include this as a Supporting Information file with your manuscript. Note that if your paper is accepted for publication, this checklist will be published with your article in the supporting information files. Please ensure that you reference the checklist in the main body of your manuscript. We suggest adding a subsection ‘Inclusivity in global research’ to your Methods section and adding the following sentence: “Additional information regarding the ethical, cultural, and scientific considerations specific to inclusivity in global research is included in the Supporting Information (SX Checklist)”

The questions have been designed to be applicable to a wide range of study types, and there are subsections for both human subjects research and non-human subjects research. If any of the questions are not relevant to your research please mark them as “N/A” as appropriate.

**Ethical considerations, permits and authorship**

*This section is applicable to all research types.*

Provide details as to who granted permissions and/or consent for the study to take place in the Methods section of your manuscript. This should include the names of **all** ethics boards, governmental organizations, community leaders or other bodies that provided approval for the study. If individuals provided approval refer to these people by their role or title but do not list their name(s).

Reported on page numbers: 39-40

If there were any deviations from the study protocol after approval was obtained please provide details of these changes in the Methods section of your manuscript.
Did this study involve local collaborators that are residents of the country where the research was conducted or members of the community studied? If you do not have any authors from said communities, please provide an explanation for this below.

Reported on page number: not applicable

Yes

Everyone listed as an author should meet PLOS’ criteria for authorship and all individuals who meet these criteria should be included in the author byline, rather than the acknowledgements. For further information please see the journal’s Authorship Policy.

**Human subjects research (e.g. health research, medical research, cross-cultural psychology)**

Did you obtain written informed consent from a representative of the local community or region before the research took place? How did you establish who speaks for the community? Details of written informed consent obtained from study participants should be reported separately in the Methods section of your manuscript.

This paper describes the protocol for a trial which, including the investigational product, has been reviewed and approved by the Ghana Food and Drug Authority (FDA, clinical trial certificate number FDA/CT/2213), the Ghana Health Service Ethical Review Committee (GHS- ERC) (GHSERC ID: 024/11/21), and the UCD, USA, Institutional Review Board (IRB) Human Subjects Committee (IRB ID: 1837253). The trial was registered on ClinicalTrials.gov (NCT05178407) and the Pan-African Clinical Trial Registry (PACTR202206868437931). Any protocol modifications will be submitted to the FDA, GHS-ERC, and UCD IRB for review, and the trial registries will be updated with approved modifications. We will seek permission from the Regional (Northern Region) and District (Tolon and Kumbungu) Directors of Health of Services as well as community leaders before recruiting participants into the study. With the assistance of the local health personnel, we will then hold general informational meetings with community leaders of each of the communities in the catchment area about the objectives, methods and possible benefits of the study for the study participants and communities.

The Regional (Northern Region) and District (Tolon and Kumbungu Districts) Health Administrations were informed about the project on a regular basis throughout pilot research conducted in 2020-2022, and these interactions will continue through trial implementation and results dissemination. We obtained permission from the Paramount Chief of the Tolon Traditional Area and The Paramount Chief of the Kumbungu traditional area to carry out the study.

As described in the manuscript, written informed consent will be obtained from all study participants prior to their participation in data collection procedures.

How did members of the local community provide input on the aims of the research investigation, its methodology, and its anticipated outcome(s)?

The Regional (Northern Region) and District (Tolon and Kumbungu Districts) Health Administrations were informed about the project on a regular basis throughout pilot research conducted in 2020-2022, including the results from pilot work and plans for the main trial (protocol reported here). In addition, study staff were recruited from the local community to implement the pilot work and some staff provided important contributions to planning the data collection methodology (including two authors of the paper).

When engaging with the local community, how did you ensure that the informed consent documents and other materials could be understood by local stakeholders?

Informed consent documents were initially developed in English by study team members, including members from Ghana. Consent documents were then translated to the local language, Dagbani, by study staff (members of the recruitment team). Disagreements about proper translation were discussed until consensus was reached. Although Dagbani is not typically used as a written language, a written version of the participant information sheet (consent form) was created in Dagbani as a reference for the recruitment team.

As required by the Ghana Health Service Ethics Review Committee, the informed consent was obtained in the presence of a literate witness if the study participant was illiterate. The literate witness subsequently signed the following ‘**Statement of Witness**’: ‘I was present when the purpose and contents of the Participant Information Sheet was read and explained satisfactorily to the participant in the language he/she understood (Dagbani). I confirm that he/she was given the opportunity to ask questions/seek clarifications and same were duly answered to his/her satisfaction before voluntarily agreeing to be part of the research.’

In addition, an informed consent quiz is included as part of the consent process to confirm the participant’s understanding of the study procedures.

Will the findings of the research be made available in an understandable format to stakeholders in the community where the study was conducted (e.g. via a presentation, summary report, copies of publications, etc.)? Please provide details of how this will be achieved.

Dissemination workshops will be held with national stakeholders in Ghana, and with regional and district stakeholders in the study area. Study results will also be disseminated through the network of bouillon-related partners and stakeholders associated with this project, with particular focus on the West Africa region. Specific dissemination activities will be informed by discussions of the trial Dissemination Advisory Group, convened by University of Ghana, UCD, and Helen Keller International, and consisting of representatives from academia and the public sector in Ghana, Burkina Faso, Senegal, and Nigeria. Publications, Statistical Analysis Plans, and other study resources will be posted on Open Science Framework (https://osf.io/t3zrn/).

**Non-human subjects research using specimens/ animals collected as part of the study, or those housed in archival collections. Examples include archaeology, paleontology, botany and zoology.**

Did the permission you obtained from a local authority to perform the study include an agreement on access to outputs and benefit sharing? This may include procedures to enable fair distribution of the benefits and resources arising from the research performed. Please include any details of Prior Informed Consent and Benefit Sharing Agreements obtained. These may be required by field-specific regulations, for example the Convention on Biological Diversity (CBD) and the associated Nagoya Protocol.

Not applicable

If the material used in your study was imported, please A) provide the year it was imported and B) indicate whether permits were obtained to import/export the materials used, C) provide details of any permits obtained. If this information is not available, please indicate this.

Not applicable

If you used archival specimens, please state how the material used in your study was acquired by the institute it is held in and provide details of any permits obtained for the original excavations/ sample collection. If this information is not available, please indicate this.

Not applicable

How was the potential cultural significance of the materials collected in your study to local communities considered in your research design? Were Indigenous peoples and/or local researchers and institutions involved with archaeological excavations / collection of specimens? If so, please provide a description of their involvement.

Not applicable

If your manuscript includes photographs of human remains please indicate whether authors obtained permission from descendants or affiliated cultural communities to do so.

Not applicable
